# Association between Ovarian Endometriomas and Stage of Endometriosis

**DOI:** 10.3390/jcm13154530

**Published:** 2024-08-02

**Authors:** Shadi Seraji, Aliyah Ali, Esra Demirel, Meredith Akerman, Camran Nezhat, Farr R. Nezhat

**Affiliations:** 1 Department of Minimally Invasive Gynecologic Surgery, NYU Langone Hospital Long Island, Mineola, NY 11501, USAmakerman@northwell.edu (M.A.); 2 Center for Special Minimally Invasive and Robotic Surgery, Camran Nezhat Institute, Woodside, CA 94061, USA; camran@camrannezhatinstitute.com; 3 Stanford University Medical Center, Palo Alto, CA 94305, USA; 4 University of California San Francisco Medical Center, San Francisco, CA 94143, USA; 5 Nezhat Surgery for Gynecology/Oncology, Valley Stream, NY 11581, USA; 6 Department of Obstetrics and Gynecology, New York Presbyterian/Weill Cornell Medical Center, New York, NY 10065, USA

**Keywords:** endometriomas, endometriosis, ASRM staging, ASRM classification, minimally invasive surgery

## Abstract

**Objectives**: To determine the association between ovarian endometriomas and stage of endometriosis. **Methods:** A total of 222 women aged 18–55 years old, who underwent minimally invasive surgery between January 2016 and December 2021 for treatment of endometriosis were included in the study. Patients underwent laparoscopic and/or robotic treatment of endometriosis by a single surgeon (FRN) and were staged using the ASRM revised classification of endometriosis. Pre-operative imaging studies, and operative and pathology reports were reviewed for the presence of endometriomas and the final stage of endometriosis. Using univariate analyses for categorical variables and the two-sample *t*-test or Mann–Whitney test for continuous data, association between endometriomas, stage of endometriosis, type of endometrioma, and other patient parameters such as age, gravidity, parity, laterality of endometriomas, prior medical treatment, and indication for surgery was analyzed. **Results:** Of the 222 patients included in the study, 86 patients had endometrioma(s) and were found to have stage III–IV disease. All 36 patients with bilateral endometriomas and 70% of patients with unilateral endometriomas had stage IV disease. **Conclusions**: The presence of ovarian endometrioma(s) indicates a higher stage of disease, correlating most often with stage IV endometriosis. Understanding the association between endometriomas and anticipated stage of disease can aid in appropriate pre-operative planning and patient counseling.

## 1. Introduction

Endometriosis is an estrogen-dependent, inflammatory, whole-body condition in which endometrial tissue is found outside of the uterine cavity. It affects up to 10–15% of women and is often associated with chronic pelvic pain, infertility, distortion of pelvic anatomy, and organ dysfunction with potential for malignant transformation [1,2,3]. Although most commonly found within the pelvic cavity affecting the reproductive tract, it can also involve other organs including the gastrointestinal, urinary, and pulmonary systems among others [4]. 

Endometriosis has also been associated with cardiovascular disease, hypertension, dyslipidemia, mood disorders, and autoimmune diseases such as systemic lupus erythematosus, rheumatoid arthritis, and inflammatory bowel disease among others [5,6,7,8]. Recent studies have also observed a significant association between endometriosis and obstetric complications such as placenta previa, placental abruption, preeclampsia, preterm labor, and premature rupture of membranes [9].

A definitive diagnosis of endometriosis is made by surgery with visual inspection and biopsy of endometriotic implants. Several endometriosis classification systems have been developed for staging the severity of endometriosis based on the density of adhesions as well as the number, size and location of implants [10]. Some common classification systems include the American Society for Reproductive Medicine (ASRM) revised classification of endometriosis, Enzian classification, Endometriosis Fertility Index (EFI) score, and American Association of Gynecologic Laparoscopists (AAGL) 2021 Endometriosis Classification. 

The most widely used is the ASRM classification, which stages endometriosis based on the location and size of endometriotic implants as well as the density of adhesions. The locations included in the ASRM classification include the peritoneum, fallopian tubes, ovaries, and posterior cul-de-sac [11]. The Enzian classification was introduced to supplement the ASRM classification in describing deeply infiltrating endometriosis (DIE) within retroperitoneal structures. It includes pelvic structures within compartments and grades endometriotic implants by size within each compartment [12,13]. The Enzian criteria were further revised to the #Enzian criteria encompassing more compartments of the pelvis and now classifies lesions within the peritoneum, ovary, fallopian tube, rectovaginal space, vagina, retrocervical space, uterosacral ligaments, cardinal ligaments, pelvic side wall, rectum, and beyond the pelvis. While both the ASRM and the Enzian systems are primarily descriptive staging tools, the EFI score is focused on pregnancy rates after surgery for endometriosis [12,14]. It takes into consideration both the description of endometriotic implants and patient factors that may affect fertility outcomes including age, years of infertility, and prior conception [14]. The AAGL Endometriosis Classification is the latest introduced system based on anatomic distribution and involvement of endometriosis, aimed at staging endometriosis by surgical complexity [15]. 

Ovarian endometriomas are found in 17–44% of patients with endometriosis [16]. Although current endometriosis staging systems rely on intraoperative findings for quantification of disease severity, the presence of endometriomas may help predict the stage of disease pre-operatively. Based on the most widely used ASRM classification system, a deep ovarian endometrioma measuring > 1 cm contributes enough points to the staging score to raise the level of disease to at least stage III [11]. Endometriomas are usually not isolated findings of DIE and often exist alongside other peritoneal disease and adhesions, which add additional points and can upstage the disease further to stage IV. 

While direct implantation on pelvic structures by way of retrograde menstruation appears to explain most cases of endometriosis, other mechanisms have been suggested for the formation of ovarian endometriomas, including invasion of functional ovarian cysts by superficial endometriotic implants and celomic metaplasia of cystic epithelial inclusion cysts [17,18,19]. Mechanisms underlying the formation of ovarian endometriomas have been further explored by histologic analysis of ovarian endometriomas in a previous study [20] by F Nezhat et al., which classified ovarian endometriomas into two main subtypes—type I endometriomas which lack luteal lining and type II endometriomas which contain luteal lining. The lack of luteal lining in type I endometriomas suggests that endometriomas may be the result of deep infiltration or invagination of the ovary by superficial endometriotic implants similar to the formation of peritoneal endometriosis. In contrast, type II endometriomas, which contain luteal lining, likely originate as follicular or luteal ovarian cysts, later invaded by endometriotic tissue. 

**Figure 1:**
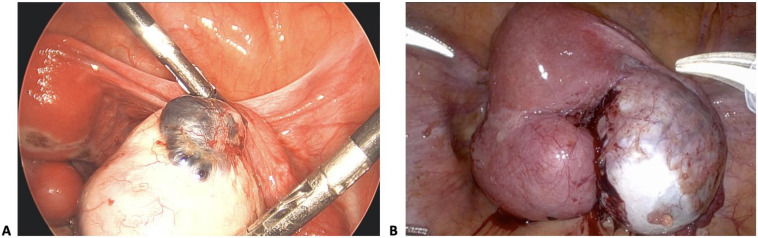
(**A**) Type I endometrioma, defined by <3 cm cyst size and adherent cyst capsule, arising from deep infiltration or invagination of superficial endometriosis implants. (**B**) Type II endometrioma, defined by >3 cm cyst size, containing gelatinous blood clots and easily delineated cyst wall, arising from physiologic functional cysts of the ovary invaded by endometriosis.

The two types of endometriomas also appear to behave differently clinically with varying degrees of capsular fibrosis and difficulty of excision. Type I endometriomas are <3 cm cysts with adherent cyst capsules, predominantly containing endometrial lining (Figure 1A). Type II endometriomas are larger, ≥3 cm cysts, containing blood-tinged or gelatinous blood clots, and have easily separated cyst capsules, containing primarily luteal lining (Figure 1B).

Previous studies have also looked at the degree of pelvic endometriosis in the presence of ovarian involvement and found an increased incidence of cul-de-sac obliteration, and bowel and ureteral endometriosis in patients with ovarian endometriomas [21,22,23]. Our study aims to correlate the presence of ovarian endometriomas with a validated and widely used endometriosis staging system by looking at the association between ovarian endometriomas and the final stage of endometriosis. The purpose of our study is to show whether the presence of ovarian endometriomas can be used as a pre-operative marker for predicting stage and anticipated severity of disease intraoperatively. Understanding the association between the presence of endometriomas and the stage of endometriosis can be helpful in pre-operative planning, anticipation of surgical challenges during surgery, appropriate surgical referrals, and pre-operative patient counseling regarding risks and benefits of surgery.

## 2. Methods

**Figure 2:**
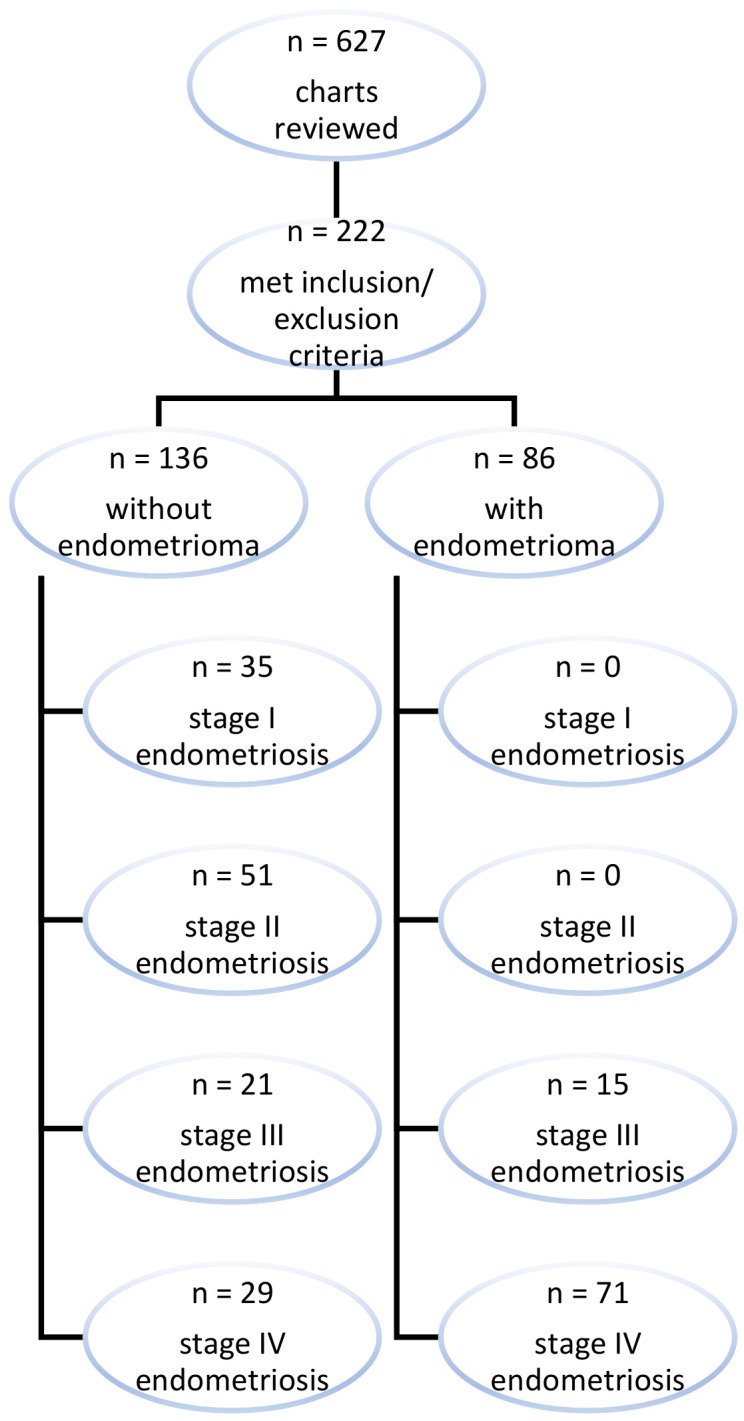
Study participants by inclusion and exclusion criteria. A total of 627 charts were reviewed and 222 subjects met inclusion/exclusion criteria. Of these, 86 patients were found to have endometrioma(s). Of the 86 patients with endometrioma(s), all had stage III–IV endometriosis.

A retrospective chart review was performed using the ICD 10 diagnosis code for endometriosis. Patients aged between 18 and 55 years old, who underwent surgery for endometriosis between January 2016 and December 2021 at a tertiary care hospital by the same surgeon and had confirmed endometriosis by pathology were included in the study. Exclusion criteria included cancer diagnosis, pregnancy, and prior bilateral oophorectomy. Of the 627 charts reviewed with a diagnosis code for endometriosis, 222 met the inclusion and exclusion criteria (Figure 2). All 222 women included in the study were treated for endometriosis by the same gynecologic surgeon with expertise in minimally invasive surgery for endometriosis (FRN).

The diagnosis of endometriosis was determined by review of operative and pathology reports. Operative reports were reviewed to determine the stage of endometriosis using the ASRM revised classification of endometriosis system based on the description and location of endometriosis implants and adhesions. The types of endometriomas (type I vs. type II) were determined from review of operative reports. Pre-operative imaging studies such as ultrasound and magnetic resonance imaging (MRI) were reviewed for presence of endometriomas to compare to intraoperative findings as described in the operative reports. Patient charts were reviewed for patient characteristics including age, gravidity, parity, and prior medical or surgical treatment for endometriosis as well as by secondary pre-operative diagnoses including pelvic pain, infertility, dysmenorrhea, dyspareunia, abnormal uterine bleeding (AUB), leiomyoma, ovarian cysts, and/or known history of endometriosis. Medical treatments were grouped into the categories of progesterone-only pill, combined oral contraceptive pills, GnRH agonist, GnRH antagonist, and other. 

## 3. Statistical Analysis 

Descriptive statistics (mean ± standard deviation for continuous variables, frequency and percent for categorical variables), univariate analyses using the chi-square test or Fisher’s exact test, as deemed appropriate for categorical variables, and the two-sample *t*-test or Mann–Whitney test for continuous data were used to compare the stage of endometriosis with the presence or absence of endometriomas. Factors which appeared to be associated with the outcome in the univariate analysis and were deemed to be clinically relevant were included in a multivariable logistic regression model. A receiver operating characteristic curve (ROC) was constructed to look at the final model’s ability to predict the outcome. A numerical measure of the accuracy of the model was obtained from the area under the curve (AUC), where an area of 1.0 signifies near-perfect accuracy, while an area of less than 0.5 indicates that the model is worse than flipping a coin. The following was used as a guide for AUC: 0.9–1.0 Excellent, 0.8–0.9 Very good, 0.7–0.8 Good, 0.6–0.7 Average, and 0.5–0.6 Poor. The Hosmer and Lemeshow Goodness-of-Fit test was also used to test how well the model fits the data. 

A result was considered statistically significant at the *p* < 0.05 level of significance. All analyses were performed using SAS version 9.4 (SAS Institute, Cary, NC, USA).

## 4. Results

Of the 222 patients analyzed, 86 were found to have endometrioma(s) and all patients with endometrioma(s) had stage III–IV endometriosis. There was a statistically significant difference between endometrioma laterality and stage of endometriosis (Table 1). All patients with bilateral endometriomas had stage IV disease and 66.67% of those with left-sided endometriomas had stage III disease (*p* = 0.0001). There was no statistically significant difference between size of endometriomas and stage of endometriosis whether right-sided (*p* = 0.8741) or left-sided (*p* = 0.3964).

The presence of endometriomas was also analyzed by patient characteristics including age, gravidity, parity, and prior medical or surgical treatment for endometriosis as well as by secondary pre-operative diagnoses, including pelvic pain, infertility, dysmenorrhea, dyspareunia, abnormal uterine bleeding (AUB), leiomyoma, ovarian cysts, and/or known history of endometriosis (Table 2). There was no significant difference for presence or absence of endometriomas based on patient age, gravidity, parity, or prior medical treatment. There was only a significant difference noted between presence of endometriomas and pre-operative diagnosis of dysmenorrhea (*p* = 0.0497) and leiomyoma (*p* = 0.0010), but there was no significant association between other indications for surgery. 

**Figure 3:**
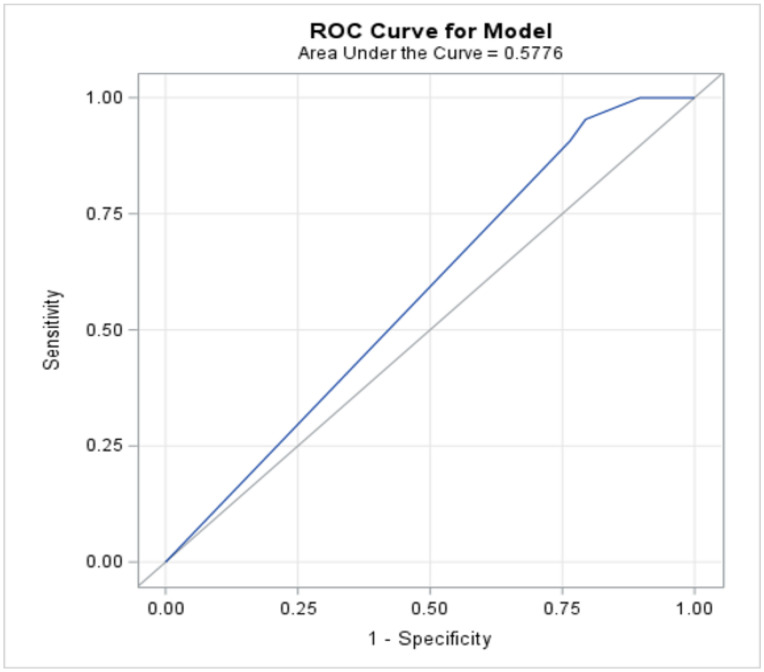
Receiver operating characteristic curve (blue line) for predictive accuracy of pelvic pain diagnoses grouped together (pelvic pain, dysmenorrhea, dyspareunia) for presence of endometrioma(s) showed poor predictive value with an area under the curve of 0.57.

Pelvic pain was also analyzed as a group including diagnoses of pelvic pain, dysmenorrhea, and dyspareunia together. Although there was a significant association with the presence of endometriomas when pelvic pain diagnoses were grouped together (*p* = 0.0370), a receiver operating characteristic curve (AUC 0.57) and multivariable logistic regression model (*p* = 0.4708) both showed poor predictive accuracy (Figure 3). 

Prior surgery for endometriosis or other abdominal surgeries had no significant association with presence of endometriomas (Table 2). Presence of endometriomas on pre-operative MRI and ultrasound was significantly associated with confirmed diagnosis of endometriomas, *p* = 0.0015 and *p* = 0.0079, respectively (Table 2). 

In patients with a prior history of surgery for endometriosis (*n* = 73), the mean interval from prior surgery to repeat surgery was 53.3 months. The median interval in months between surgeries decreased with increasing stage of endometriosis, with 60 months, 51 months, 24 months, and 26 months for stages 1, 2, 3, and 4 endometriosis, respectively (*p* = 0.359). 

Medical treatments were analyzed in the categories of progesterone-only pill, combined oral contraceptive pills, GnRH agonist, GnRH antagonist, and other (Table 3). The majority of the patients were treated with combined oral contraceptive pills (43.6%), followed by progesterone-only pill (19%) and GnRH agonist (19%). Patients on a longer duration of medical therapy (>12 months) had lesser stages of disease compared to those with a shorter duration of medical therapy (<3 months) (Table 4). In patients who were treated with medical therapies for >12 months, 73.3% had stage I disease, compared to 75%, 38.9%, and 20.8% who had stage II, III, and IV disease, respectively. In patients who were treated with medical therapies for <3 months, 58.5% had stage IV disease, compared to 38.9%, 20.8%, and 13.3% who had stage III, II, and I disease, respectively. 

Stage of endometriosis was significantly associated with the presence of endometriomas (*p* ≤ 0.0001, Table 2). All patients with endometrioma(s) had stage III–IV disease (100%), whereas the majority of patients with no endometriomas had stage I–II disease (63.24%). 

Stage of endometriosis was also analyzed by the type of endometrioma present. The majority of type I (71.43%) and type II (88.52%) endometriomas were associated with stage IV endometriosis (Table 5). 

## 5. Discussion

Endometriosis can result in dense adhesions and fibrosis in the pelvis that distort normal abdomino-pelvic anatomy. Endometriosis implants are commonly found on pelvic structures such as ovaries, fallopian tubes, pelvic peritoneum, sigmoid colon, and the Pouch of Douglas. The location of these implants is thought to be gravity dependent based on the commonly accepted theory of retrograde menstruation as the pathogenesis of endometriosis [24]. 

While direct implantation on pelvic peritoneum and other pelvic structures by way of retrograde menstruation appears to explain most cases of endometriosis, other mechanisms have been suggested for formation of ovarian endometriomas. In 1921, John Sampson suggested endometriomas may arise from invasion of functional ovarian cysts by superficial endometriotic implants [17]. Other theories have proposed celomic metaplasia of cystic epithelial inclusion cysts commonly found in ovaries [18,19]. 

A clinical and histologic analysis of ovarian endometriomas by F. Nezhat et al. demonstrated that the majority of endometriomas histologically arise from corpus luteum cysts. This histopathologic analysis classifies endometriomas into types I and II based on the presence or absence of endometrial lining and the degree of adherence of the cyst capsules (Figure 1A,B). The finding that type I endometriomas lack luteal lining suggests that these endometriomas may not be arising from functional cysts as previously proposed by Sampson, but likely the result of deep infiltration or invagination of the ovary by superficial endometriotic implants and reactive fibrosis. In contrast, type II endometriomas, which contain luteal lining, supports the theory that these cysts likely originate as follicular or luteal ovarian cysts, later invaded by endometriotic tissue. Varying degrees of capsular fibrosis and difficulty of surgical excision between the two types of endometriomas are clinically important, especially in surgical evaluation of infertility patients with higher concern for risk of ovarian compromise. The majority of patients in this study had type II endometriomas (74%). 

The ASRM revised classification of endometriosis system measures severity of disease based on location, size, depth of lesions, and density of adhesions [11]. Endometrioma is considered a form of DIE. In our study, the majority of patients with endometriomas were found to have stage IV disease (86.56%, *p*-value < 0.0001) with a significant association between presence of endometriomas and stage of disease. Although it is already accepted that a deep ovarian endometrioma measuring > 1cm contributes enough points in the ASRM scoring system to upstage disease to at least stage III, our study shows that the actual stage is often more severe and correlates to stage IV. 

**Figure 4:**
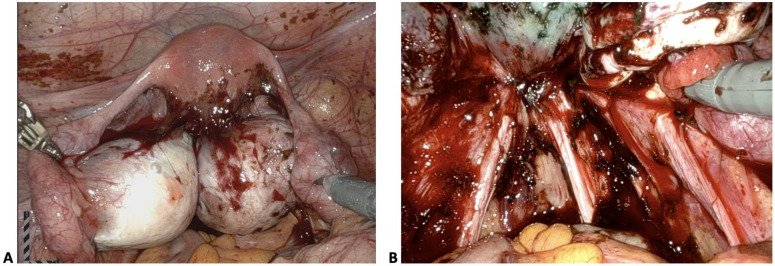
(**A**) Bilateral endometriomas with kissing ovaries and dense adhesions to posterior uterus. (**B**) Endometriotic lesions along bilateral uterosacral ligaments and deep infiltrating endometriosis of posterior cul-de-sac in a patient with bilateral ovarian endometriomas.

The higher stage of endometriosis with the presence of endometriomas is likely due to deeply infiltrative nature of ovarian endometriomas, which are rarely isolated findings in endometriosis. Patients who have ovarian endometriomas tend to have other forms of DIE such as kissing ovaries, and dense adhesions between other pelvic organs and structures such as the rectosigmoid colon, pelvic side wall, and uterosacral ligaments (Figure 4) [10]. 

DIE is generally associated with a greater degree of adhesions and fibrosis with resulting distortion of anatomy. Excision of DIE can be difficult with a greater risk of unanticipated injuries, longer operating times, and increased blood loss. Treatment of endometriomas can also be challenging, especially in infertility patients [25]. Excision of endometriomas may result in potential damage or unintentional compromise of surrounding healthy ovarian tissue, thus decreasing ovarian reserve [25,26]. 

Our results are consistent with previous studies demonstrating increased severity of endometriosis when the ovaries are involved. In a previous study by Redwine et al., which looked at disease severity with ovarian involvement, 21.7% of patients with ovarian endometriosis had complete cul-de-sac obliteration compared to only 5.8% in those without ovarian involvement [21]. The study also showed an increased incidence of intestinal endometriosis in those with ovarian involvement, especially with ovarian cysts larger than 1 cm. 

In another study by Araujo et al., patients with unilateral endometriomas were found to have more severe DIE, especially of the bowel, vagina, and ureter [22]. The study also found that endometriomas tend to be slightly more common on the left side (54.8%) compared to the right side (45.2%). This was also true in our study, where 56% of the 50 unilaterally identified endometriomas were left-sided compared to 44% right-sided. Left-sided predisposition of endometriomas has been theorized to be due to the presence of the sigmoid colon on the left side, which restricts the flow of peritoneal fluid in the left hemipelvis, allowing a greater opportunity for implantation and infiltration of endometrial fragments to the left ovary and surrounding tissues [23]. 

Although previous studies have similarly demonstrated a greater degree of disease with ovarian involvement of endometriosis, our study is the first to correlate it with a staging system to our knowledge. In the current study, we show that unilateral and bilateral endometriomas are significantly correlated with stage III–IV endometriosis. While it is hard to detect all potential implants of endometriosis on pre-operative imaging such as ultrasound and MRI, endometriomas can often be reliably identified on these imaging studies by their characteristic features of homogeneous low-level echoes or a ground-glass opacities [27]. Pre-operative detection of endometriomas by imaging prior to surgery and anticipation of stage III–IV disease can heighten a surgeon’s caution and anticipation for surgical challenges that may lie ahead. 

The pre-operative predictive value of ovarian endometriomas for stage of endometriosis is also important in appropriate pre-operative counseling of patients about the potential complications and outcomes of surgery. Given the possible complications that can occur with higher stages of endometriosis, some patients may choose to opt for medical treatments or referral to the appropriate surgical experts. 

Our study utilized the ASRM revised classification of endometriosis due to it being both the most widely used classification in describing the stage of endometriosis and the system that we currently use in practice. It accurately and simply describes mild-to-severe endometriosis and its simplicity also serves as a benefit when explaining endometriosis to patients. Correlating our results with the most widely utilized classification system allows better communication and wider application of our findings for diagnosis and treatment. 

There remain limitations to the utility of the ASRM revised classification, however, as it does not fully detail the extent of DIE across all organs and systems. Newer classification systems, such as the #Enzian, and the AAGL 2021 endometriosis classification divide the pelvis into multiple compartments and even include implants beyond the pelvis [15,28]. These newer classifications provide a more comprehensive description of pelvic and peritoneal disease to better describe extent of endometriosis. While these classifications may better classify DIE, they are not as widely accepted or used in practice currently. As these systems become more widely used, there may be benefit in extending our study to include these newer classification systems as associated with endometriomas in the future. 

Various non-invasive imaging techniques have also been proposed over the years such as the Ultrasound-Based Endometriosis Staging System (UBESS) to allow non-surgical evaluation of the extent of pelvic endometriosis [29]. Other imaging modalities include MRI, and less commonly computed tomography (CT), barium enema, and intravenous urography [30]. While these tools are useful diagnostic modalities in the evaluation of DIE, the accuracy and performance of these techniques are operator dependent. Endometriomas are often the most accurately described form of endometriosis on imaging, therefore we have chosen to focus on endometriomas as associated with severity of disease. 

## 6. Conclusions

Endometriomas are a form of DIE associated with higher stages of endometriosis. Our study shows unilateral endometriomas are associated with at least stage III disease and bilateral endometriomas are associated with stage IV disease. This is likely due to the fact that an endometrioma is rarely an isolated occurrence and often associated with other forms of DIE. This can make surgical treatment a challenge, with a more difficult and lengthy surgery as well as higher risk of complications. 

Currently, surgery is the only definitive way to diagnose endometriosis. While current screening tests and imaging studies are limited in the diagnosis of endometriosis, ovarian endometriomas can often be reliably identified on ultrasound and MRI pre-operatively. Understanding the association of endometriomas with the expected level of disease can aid in appropriate surgical planning. The pre-operative predictive value of endometriomas for severity of disease can both serve as an adjunct to the current ASRM classification of endometriosis and aid in appropriate patient counseling and referral to appropriate expertise. 

## Figures and Tables

**Table 1:** Association of laterality and size of endometrioma(s) with stage of endometriosis.

		Stage of Endometriosis		
		3 (*n* = 15)	4 (*n* = 71)	*p*-Value
Laterality of endometrioma	Left	10 (66.67%)	18 (25.35%)	0.0001
	Right	5 (33.33%)	17 (23.94%)	
	Bilateral	0 (0%)	36 (50.7%)	
Size of right endometrioma (cm)	≤3 cm	1 (20%)	19 (35.85%)	0.8741
	3–6 cm	3 (60%)	21 (39.62%)	
	6–9 cm	1 (20%)	10 (18.87%)	
	≥10 cm	0 (0%)	3 (5.66%)	
Size of left endometrioma (cm)	≤3 cm	7 (70%)	21 (38.89%)	0.3964
	3–6 cm	2 (20%)	21 (38.89%)	
	6–9 cm	1 (10%)	9 (16.67%)	
	≥10 cm	0 (0%)	3 (5.56%)	

**Table 2:** Univariable analyses for presence/absence of endometrioma(s) by patient characteristics, secondary diagnoses, prior surgery for endometriosis, prior abdominal surgeries, and pre-operative imaging.

		Presence of Endometriomas		
		No (*n* = 136)	Yes (*n* = 86)	*p*-Value
Age		36.6 ± 8.4	37.2 ± 7.6	0.5675
Gravidity		1.32 ± 1.74	1.05 ± 1.45	0.2411
Parity		0.67 ± 0.95	0.52 ± 0.84	0.2865
Medical treatment prior to surgery		74 (54.41%)	48 (55.81%)	0.8379
Indication for surgery	Pelvic pain	83 (61.03%)	47 (54.65%)	0.3473
	Infertility	31 (22.79%)	29 (33.72%)	0.0741
	Dysmenorrhea	72 (52.94%)	57 (66.28%)	**0.0497**
	Dyspareunia	47 (34.56%)	33 (38.37%)	0.5643
	Abnormal uterine bleeding	81 (59.56%)	48 (55.81%)	0.5817
	Other ovarian cyst	8 (5.88%)	8 (9.3%)	0.3371
	Leiomyoma	28 (20.59%)	4 (4.65%)	**0.0010**
	History of endometriosis	41 (30.15%)	28 (32.56%)	0.7053
General pelvic pain (pelvic pain, dysmenorrhea, and dyspareunia)		118 (86.76%)	82 (95.35%)	**0.0370**
Prior surgery for endometriosis		52 (38.24%)	30 (34.88%)	0.6142
Prior abdominal surgery		60 (44.12%)	36 (41.86%)	0.7409
Pre-operative imaging	MRI	48 (35.29%)	49 (56.98%)	**0.0015**
	Ultrasound	98 (72.06%)	47 (54.65%)	**0.0079**
Stage of endometriosis	1	35 (25.74%)	0 (0%)	**<0.0001**
	2	51 (37.5%)	0 (0%)	
	3	21 (15.44%)	15 (17.44%)	
	4	29 (21.32%)	71 (82.56%)	

**Table 3:** Types and duration of medical treatments prior to surgery.

Duration of Medical Management Prior to Surgery	Types of Medical Management					
	GnRH Agonist	GnRH Antagonist	Combined Oral Contraceptive Pills	Progesterone only	Other	Total
≤3 mo	14	11	11	6	3	45
>3 but ≤6 mo	5	0	2	3	2	12
>6 but ≤12 mo	1	0	3	2	0	6
>12 mo	1	0	32	10	4	47
Total	21	11	48	21	9	110

**Table 4:** Duration of medical treatment prior to surgery and final stage of endometriosis.

Duration of Medical Management Prior to Surgery	Stage of Endometriosis				
	1	2	3	4	Total
≤3 mo	2	5	7	31	45
>3 but ≤6 mo	2	0	3	7	12
>6 but ≤12 mo	0	1	1	4	6
>12 mo	11	18	7	11	47
Total	15	24	18	53	110

**Table 5:** Association between type of endometrioma and stage of endometriosis.

Type of Endometrioma	Stageof Endometriosis		
	3	4	Total
Type I (<3 cm)	6	15	21
Type II (≥3 cm)	7	54	61

## Data Availability

The raw data supporting the conclusions of this article are available on request from the corresponding author.
